# Nitrogen Removal Efficiency for Pharmaceutical Wastewater with a Single-Stage Anaerobic Ammonium Oxidation Process

**DOI:** 10.3390/ijerph17217972

**Published:** 2020-10-30

**Authors:** Lushen Zuo, Hong Yao, Huayu Li, Liru Fan, Fangxu Jia

**Affiliations:** School of Civil Engineering, Beijing Jiaotong University, Beijing 100044, China; zls19900606@163.com (L.Z.); lihuayuchina@163.com (H.L.); hagongflr@126.com (L.F.); fxjia@bjtu.edu.cn (F.J.)

**Keywords:** pharmaceutical wastewater, nitrogen removal, single-stage ANAMMOX, chlortetracycline, COD

## Abstract

A single-stage anaerobic ammonium oxidation (ANAMMOX) process with an integrated biofilm–activated sludge system was carried out in a laboratory-scale flow-through reactor (volume = 57.6 L) to treat pharmaceutical wastewater containing chlortetracycline. Partial nitrification was successfully achieved after 48 days of treatment with a nitrite accumulation of 70%. The activity of ammonia oxidizing bacteria (AOB) decreased when the chemical oxygen demand (COD) concentration of the influent was 3000 mg/L. When switching to the single-stage ANAMMOX operation, (T = 32–34 °C, DO = 0.4–0.8 mg/L, pH = 8.0–8.5), the total nitrogen (TN) removal loading rate and efficiency were 1.0 kg/m^3^/d and 75.2%, respectively, when the ammonium concentration of the influent was 287 ± 146 mg/L for 73 days. The findings of this study imply that single-stage ANAMMOX can achieve high nitrogen removal rates and effectively treat pharmaceutical wastewater with high concentrations of COD (1000 mg/L) and ammonium.

## 1. Introduction

Pharmaceutical wastewater is rich in organic pollutants, ammonia (NH_3_), and multiple inhibitory materials that can adversely impact the performance of wastewater treatment. In recent years, effluent standards for wastewater have become stricter in China. The ammonium (NH_4_^+^) concentration of most pharmaceutical wastewater effluent does not meet the Chinese discharge standard due to the high NH_4_^+^ concentration of influent and the limited removal efficiency of current treatment processes, including nitrification–denitrification.

Compared to the conventional nitrification–denitrification process, anaerobic ammonium oxidation (ANAMMOX) is recognized as a cost-efficient nitrogen (N) removal process with a shorter NH_3_ removal cycle that directly converts NH_4_^+^ to N gas using nitrite (NO_2_^−^) as an electron acceptor [1,2,3]. Nitrite is unstable in wastewater because it is easily oxidized to nitrate (NO_3_^−^), and ANAMMOX cannot be applied directly to wastewater [4]. Therefore, it is necessary to combine ANAMMOX with partial nitrification in a single reactor, which is known as the single-stage ANAMMOX process [5,6]. In the single-stage ANAMMOX process, NH_4_^+^ is partially oxidized to NO_2_^−^ by ammonia oxidizing bacteria (AOB), after which, NO_2_^−^ and residual NH_4_^+^ are converted to N gas by anaerobic ammonium-oxidizing bacteria [7]. This process has many advantages, such as no need for external carbon, low excess sludge production and a high treatment efficiency in treating industrial wastewater with high ammonium [8]. The single-stage ANAMMOX process has been successfully applied for treating landfill leachate that contains a high NH_4_^+^ concentration [9].

In the current process of producing antibiotics in China, the chemical oxygen demand (COD) for wastewater typically ranges from 500 mg/L to 1000 mg/L. High COD values and a high carbon/nitrogen (C/N) ratio are the main inhibitors of single-stage ANAMMOX for treating pharmaceutical wastewater [10]. This is because they can both inhibit the growth of autotrophic bacteria that are responsible for N removal. Therefore, it is necessary to study the effect of high COD levels on AOB in the single-stage ANAMMOX process [11].

In this study, an integrated biofilm–activated sludge single-stage ANAMMOX system was initiated by increasing the influent N-loading rate and varying the C/N ratio. The purpose of this study is to investigate the performance of a single-stage ANAMMOX process in treating pharmaceutical wastewater with a high COD concentration and to determine whether the NH_4_^+^–N concentration of effluent meets the Discharge Standard of Water Pollutants for the Pharmaceutical Industry in China (GB 21903-2008). This research could provide some insight into the efficiency of single-stage ANAMMOX with respect to the theoretical basis for upgrading the quality of pharmaceutical wastewater in China.

## 2. Materials and Methods

### 2.1. Reactor and Operation Conditions

**Figure 1:**
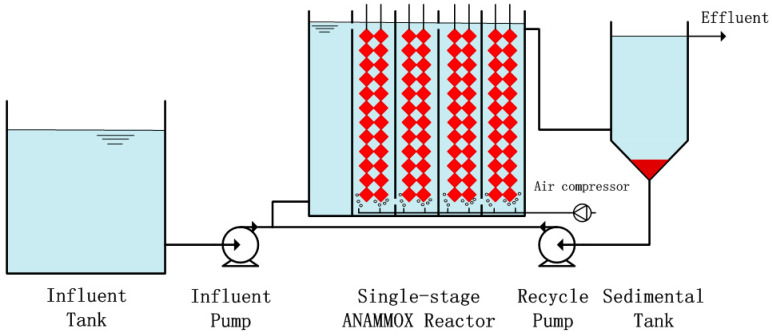
Flow chart of the single-stage anaerobic ammonium oxidation (ANAMMOX) process. The reactor was composed of two rows, and the influent and recycled water were introduced using peristaltic pumps.

In this work, a single-stage ANAMMOX process was operated for 121 days and divided into eight stages (Figure 1). This study was carried out in a laboratory-scale reactor (0.65 m × 0.31 m × 0.50 m) with a total volume of 57.6 L. The wastewater was pumped into the reactor with 1.6 L/h inflow. The hydraulic retention time was kept at 36 h in whole experiment. Moreover, the reflux ratio was 150% to make most of sludge return to reactor. The temperature of the reactor was controlled at 33 °C ± 1 °C using heating rods. Thermal insulation material was used to provide a constant temperature and to isolate bacteria from the inhibiting influence of light. The pH in the reactor was maintained in the range of 8.0–8.5 by adding KHCO_3_.

### 2.2. Seed Sludge and Experimental Setup

This study consisted of two periods: the partial nitrification period and the single-stage ANAMMOX period. The partial nitrification process was initiated first by introducing 10 L of nitrification sludge from a cyclic activated sludge system (CASS). This CASS is located at a wastewater treatment plant that treats chlortetracycline (CTC) wastewater in Inner Mongolia, which was introduced into the reactor. The partial-nitrification biomass concentration of the sludge (as the content of mixed liquor suspended solids (MLSS) was 5 kg/L. The hydraulic retention time (HRT) was 32 h. At the beginning of this period, the feed was the diluted effluent of the aerobic process from the same plant. The NH_4_^+^–N and COD concentrations of the aerobic effluent were 100–200 mg/L and 500–1000 mg/L, respectively, and the C/N ratio was approximately 4.2. The influent was then changed to a mixture of aerobic and anaerobic effluents from the treated CTC wastewater. The NH_4_^+^–N and COD concentrations of the anaerobic effluent were 510 ± 55 mg/L and 2447 ± 422 mg/L, respectively. The partial nitrification period was divided into four stages by different mixture rates of aerobic and anaerobic effluents (Table 1).

In order to initiate the single-stage ANAMMOX process, ANAMMOX biomass samples were taken from an ANAMMOX up-flow column reactor in the form of sponge and added into the single-stage system. Moreover, the nitrogen removal rate of the seed sludge reactor was 0.52 ± 0.24 kg/m^3^/d. The seed sludge reactor was used for treating the sludge fermentation liquid with 450 ± 255 mg/L NH4^+^–N of typical domestic wastewater treatment plant in Beijing. The biomass consisted of *Planctomycete*-like ANAMMOX bacteria and the filling rate was 30%. In this period, the influent of the reactor was the effluent of the anaerobic process. The average COD, NH4^+^–N, SO_4_^2−^, and CTC concentrations of the influent were 782 ± 162 mg/L, 143 ± 66 mg/L, 232 ± 87 mg/L, and 0.9 ± 0.6 mg/L, respectively. According to different dilution times of the anaerobic effluent, the single-stage ANAMMOX period was divided into three stages (Table 1).

### 2.3. Batch Test

In the partial nitrification period, a series of batch tests were performed to determine the activity of AOB. The batch assays were conducted in conical flasks with an effective liquid volume of 250 mL. Influent and biomass samples were taken from the reactor at the end of each stage and the pH was adjusted to 8.0–8.5 using KHCO_3_. Finally, the flasks were placed in a constant temperature shaking incubator at 35 °C ± 1 °C and 180 rpm for 32 h. The NH_4_^+^–N and COD concentrations were analyzed at regular intervals using syringe filters to take samples from each flask.

### 2.4. Chemical Analysis

Nitrogen removal efficiency was evaluated by quantifying the NH_4_^+^–N, NO_2_^−^–N, and NO_3_^−^–N concentrations of influent and effluent samples. All samples were collected on a daily basis and analyzed immediately or stored in a refrigerator at 4 °C awaiting analysis. The determinations of COD, NH_4_^+^–N, NO_2_^−^–N, and NO_3_^−^–N concentrations were performed according to the standard methods [12]. A digital pH meter was used to determine pH potentiometrically. The dissolved oxygen (DO) concentration was measured with a digital DO meter (SAXIN, X751).

## 3. Results and Discussion

### 3.1. Partial Nitrification Period

**Figure 2:**
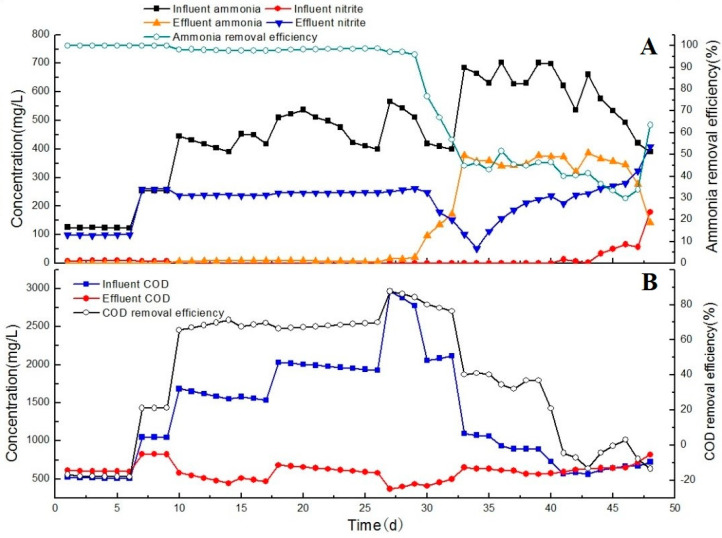
Nitrogen removal performances in partial nitrification process, showing the (**A**) ammonia removal efficiency, and (**B**) chemical oxygen demand (COD) removal efficiency.

During the partial nitrification process, the activity of AOB was influenced by the influent COD concentration (Figure 2). During the first 7 days, the influent COD and NH_3_ loading rate (as N) were 0.4 kg/m^3^/d and 0.1 kg/m^3^/d respectively. When the system was fed with the effluent of the CASS process, more than 90% of NH_3_ was transformed to NO_2_^–^ after the first 7 days. The NH_3_ conversion rate increased rapidly to ~0.07 kg/m^3^/d (as N). Thus, the partial nitrification process was successfully initiated. Between days 7 and 26, the influent was changed to a mixture of aerobic and anaerobic effluent of the treated CTC wastewater. As the mixture ratio was gradually increased, the COD and NH_4_^+^–N concentrations increased to 2000 mg/L and 500 mg/L, respectively. When the COD concentration was 500 mg/L, 1000 mg/L, 1500 mg/L, and 2000 mg/L, and the NH_4_^+^–N concentration was 300 mg/L ± 100 mg/L, the NH_4_^+^–N concentration of the effluent was <50 mg/L. More than 80% of NH_3_ was transformed to NO_2_^−^ at a conversion load of 0.3–0.4 kg/m^3^/d (as N) when the C/N ratio was approximately 3–4; hence, the activity of AOB was not inhibited at a low COD concentration.

The NH_3_ conversion rate remained stable until the COD loading rate reached 2.5 kg/m^3^/d and the C/N ratio was 6. By day 27, the ratio of aerobic/anaerobic influent was 2:1, and the COD concentration was 3200 mg/L ± 150 mg/L. The NH_4_^+^–N concentration of the effluent rapidly increased to 400 mg/L. The NH_3_ conversion rapidly decreased to 10% at a high COD concentration (3000 mg/L) and the NH_3_ transforming rate was <0.1 kg/m^3^/d (as N). This result suggests that the activity of AOB could be significantly constrained by a high COD loading rate, and that a COD concentration of 3000 mg/L could inhibit the partial nitrification process.

In order to recover the nitrification process, the mixture rate was gradually decreased. At the end of this stage, the influent mixture ratio returned to 1:1 and the effluent ammonium was decreased to 185 mg/L, and the nitrogen removal efficiency recovered to 67%. By day 48, the COD and NH_4_^+^–N concentrations of the influent were 500–700 mg/L and 500–600 mg/L, respectively. Approximately 70% of NH_3_ was transformed to NO_2_^−^. The activity of AOB gradually recovered to a final NH_3_ conversion rate of 0.35 kg/m^3^/d when the COD concentration of the influent was 1000 mg/L (COD loading of 0.5 kg/m^3^/d). These results indicate that the inhibition of the activity of AOB due to the COD was reversible.

Other studies reported that when a partial nitrification system was operated at a C/N ratio of 2, AOB was effective for NH_3_ removal, while altering the C/N ratio to 5 resulted in a 50% reduction in the nitrification removal [13,14]. Another study reported that the optimum COD concentration for nitrogen removal from pharmaceutical wastewater was approximately 500 mg/L when NH_3_ levels were ~100 mg/L [15]. The findings for the partial nitrification system investigated in the present study suggests that high NH_3_ removal rates can be achieved when the COD concentration, NH_4_^+^–N concentration, and C/N ratio of influent are high.

### 3.2. Batch Test

**Figure 3:**
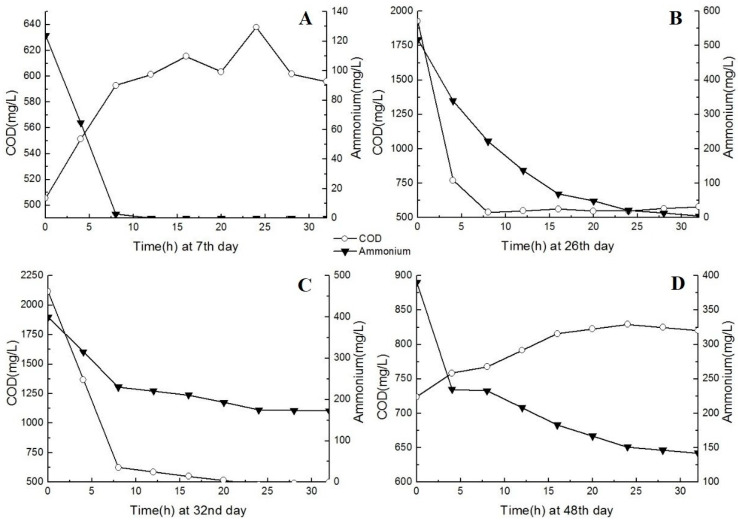
Relationships between ammonia and the chemical oxygen demand (COD) concentration for batch tests in different stages, showing (**A**–**D**).

The feasibility of treating pharmaceutical wastewater during the partial nitrification period was initially assessed by batch tests. Biomass samples were taken from the reactor at the end of each stage (days 7, 26, 32, and 48). The biomass concentration was set at 5 g/L (as MLSS) and the HRT was set to 32 h. The conversion rates of NH_3_ and COD were significantly affected by the different COD conditions in each stage (Figure 3).

In the first stage, NH_3_ was degraded completely within 8 h when the COD concentration of the influent was <1000 mg/L. The COD concentration of the influent continued to increase from an HRT of 0 h (500 mg/L) to an HRT of 30 h, and could not be easily degraded by microorganisms. Nearly all NH_3_ was transformed to NO_2_^−^, and this process drove the COD concentration of the effluent to exceed that of the influent. When the COD concentration of the influent increased to 2000 mg/L on day 26, more than 20 h were required to completely degrade NH_3_. This was due to the higher NH_3_ concentration of the influent and the competition for DO by AOB to remove a high COD concentration. In the third stage (day 32), NH_3_ degradation decreased rapidly to 40% when the COD concentration was >3000 mg/L. As the COD concentration of the influent increased, the degradation of the COD also increased from 0% to 70%. These results indicate that the DO in the reactor was first used to oxidize organic compounds (e.g., COD) and then to oxidize NH_3_. This agrees with the results of the second stage and indicates that heterotrophic bacteria could grow faster than AOB under a high COD concentration.

After the recovery period in the fourth stage, more than 50% of NH_3_ could be transformed to NO_2_^−^. The results of the batch test showed that a high COD concentration (>3000 mg/L) could inhibit the NH_3_ degradation rate and the activity of AOB.

### 3.3. Single–Stage ANAMMOX Period

In order to initiate the single–stage ANAMMOX process, ANAMMOX biomass samples were taken from an ANAMMOX up–flow column reactor that was packed with a sponge biomass carrier. This period could be divided into three phases over a total of 79 days.

In stage I (from day 49 to day 52), the reactor was fed with aerobic effluent from treated CTC wastewater. The COD and NH_4_^+^–N concentrations of the influent were 700 mg/L and 370 mg/L, respectively. At the beginning of this stage, the ammonium removal rate increased rapidly. When the influent wastewater was changed to the diluted anaerobic effluent of the CTC wastewater, the NH_4_^+^–N removal rate reduced to nearly zero. This was because AOB could not adapt to the change in water quality. The activity of ANAMMOX was also inhibited by the high concentration of NO_2_^−^–N in the reactor in this period. Moreover, ANAMMOX bacteria were not the predominant bacteria, and the total N (TN) removal was still <30%. Gradually, AOB adapted to the water quality and the N removal rate increased steadily. At the end of the first stage, the NH_4_^+^–N removal rate gradually increased and the NO_2_^−^–N concentration in the effluent was close to zero. The single–stage ANAMMOX process was successfully initiated with the NH_4_^+^–N, NO_2_^−^–N, and NO_3_^−^–N concentrations of the effluent at 80 mg/L ± 20 mg/L, 10 mg/L ± 5 mg/L, and 20 mg/L ± 10 mg/L, respectively. However, when the ammonium concentration of the influent was low, the N loading rate was limited to 0.23 kg/m^3^/d. However, the ratio of AOB/NH_4_^+^–N transformation was higher than that of ANAMMOX/NH_4_^+^–N transformation because ANAMMOX bacteria did not completely adjust to the anaerobic influent wastewater.

Increasing the N loading rate and N removal rate were the main objectives in phase II, which was from day 53 to day 92. At the beginning of the second stage, the influent of the reactor was the diluted anaerobic effluent of treated CTC wastewater. The COD and NH_4_^+^–N concentrations of the influent were 510 mg/L ± 2 mg/L and 160 mg/L ± 10 mg/L, respectively. When the TN removal rate reached 75–80%, the COD and NH_4_^+^–N concentrations of the influent were increased by reducing the dilution ratio of the anaerobic effluent. At the end of this phase, the dilution ratio decreased to 1:1.33, and the COD and NH_4_^+^–N concentrations of the influent were 600 mg/L and 450 mg/L, respectively. The NH_4_^+^–N and NO_2_^−^–N concentrations of the effluent were 115 mg/L ± 35 mg/L and <10 mg/L, respectively. The NH_3_–N removal rate and TN removal were 80% and 75%, respectively. In this stage, the TN concentration of the influent gradually increased, while the TN removal remained at 60–70%. The COD concentration of the influent ranged from 400 mg/L to 1000 mg/L, whereas that of the effluent was stable at 400–500 mg/L. It could be inferred from the results that ANAMMOX bacteria became the predominant bacteria and were unaffected by the COD of the influent. Meanwhile, the COD removal was approximately 30%, which indicates that there were also some heterotrophic bacteria and denitrifying bacteria in the reactor.

**Figure 4:**
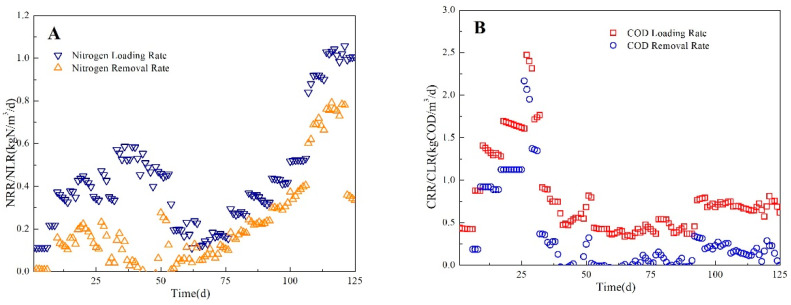
The loading rate and removal rate of ammonium (**A**) and COD (**B**) in whole experiments.

As shown in Figure 4A, from day 93 to day 123 in the third stage, the NH_4_^+^–N loading rate of the influent increased to 1.1 kg/m^3^/d (as N) when the HRT of the reactor was decreased to 16 h. The COD and NH_4_^+^–N concentrations of the influent were 800–900 mg/L and 450–650 mg/L, respectively. At the beginning, the NH_3_ concentration of the effluent reached 200 mg/L and the TN removal rate decreased slightly. After 28 days of operation, the NH_4_^+^–N, NO_2_^−^–N, and NO_3_^−^–N concentrations of the effluent were 91 mg/L ± 12 mg/L, 6 mg/L ± 5 mg/L, and 45 mg/L ± 15 mg/L, respectively. The TN removal was 75% and the N removal load was 1.0 kg/m^3^/d when the NH_4_^+^–N concentration of the influent was 300–600 mg/L. At the end of this steady operation stage, the NH_4_^+^–N and TN concentrations of the effluent were maintained at <50 mg/L and <100 mg/L, respectively, when the TN concentration of the influent was 580–650 mg/L. In this phase, the ratios of AOB/NH_4_^+^–N transformation and ANAMMOX/NH_4_^+^–N transformation rapidly approached 1:1. This means that the reactions in the reactor were dominated by partial nitrification and ANAMMOX. The TN removal was mostly associated with the partial nitrification–ANAMMOX reaction rather than conventional nitrification–denitrification reactions. As the NH_4_^+^ and COD concentrations increased, the activities of both AOB and ANAMMOX bacteria increased.

**Figure 5:**
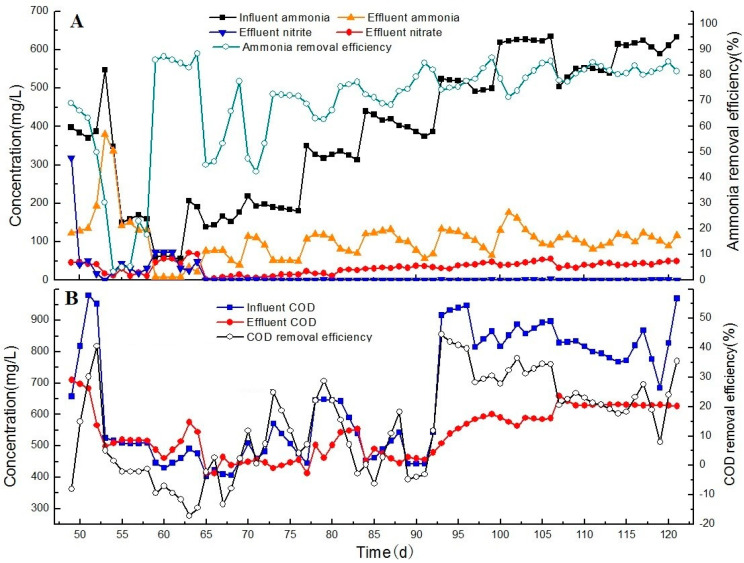
Nitrogen removal performances in the single–stage ANAMMOX process showing the (**A**) ammonia removal efficiency, and (**B**) chemical oxygen demand (COD) removal efficiency.

In the single–stage ANAMMOX period, the TN removal efficiency and TN loading rate gradually increased to 75.2% and 1.0 kg/m^3^/d, respectively, and then remained high until the end of the study (Figure 5), which agrees with previous reports [16]. This result demonstrates that influent wastewater with a COD concentration of <1000 mg/L could not restrain the activities of AOB and ANAMMOX bacteria. The single-stage ANAMMOX process could achieve high nitrogen removal efficiency when COD loading rate was lower than 1.0 kg COD/m^3^/d (Figure 4B). The maximum C/N ratio was approximately 2.0, which was higher than that reported in most studies. The final N removal load was similar to that reported for other single–stage ANAMMOX processes when treating wastewater with a low C/N ratio (≤1) (Table 2). This implies that single–stage ANAMMOX is an efficient process for treating pharmaceutical wastewater with a high COD concentration and high C/N ratio.

## 4. Conclusions

The results of this study indicate that single–stage ANAMMOX is an effective process for treating pharmaceutical wastewater with a high COD concentration. The partial nitrification process and single–stage ANAMMOX period were both successfully initiated, and achieved a high N removal efficiency. The TN removal efficiency and TN loading rate were 75.2% and 1.0 kg/m^3^/d, respectively, under the tested conditions (T = 32–34 °C, DO = 0.4–0.8 mg/L, and HRT of 16 h) when the TN concentration of the influent was 600 mg/L and that of the effluent was 150–200 mg/L. The NH_4_^+^ concentration of the effluent during the last stage of the single–stage ANAMMOX period was <50 mg/L, which met the Discharge Standard of Water Pollutants for Pharmaceutical Industry in China (GB 21903–2008). The COD and NH_4_^+^–N concentrations along with the C/N ratio were the key factors impacting this process. The activity of AOB could be significantly constrained by a high COD loading rate (>2000 mg/L). This inhibition was reversible when decreasing the COD concentration to <1000 mg/L. The activity of ANAMMOX bacteria treating the pharmaceutical wastewater was not inhibited when the COD concentration was <1000 mg/L and the C/N ratio was approximately 2. This study provides insight into the efficiency of single–stage ANAMMOX with respect to the theoretical basis for upgrading the quality of pharmaceutical wastewater in China

## Figures and Tables

**Table 1:** Different stages during the partial nitrification process and single-stage ANAMMOX process.

Stage	Time (Day)	Purpose	COD (mg/L)	NH_4_^+^–N (mg/L)	Influent
1–1	1–7	Starting up for nitrification	782 ± 162	143 ± 66	All aerobic
1–2	8–26	Increasing influent nitrogen load	1577 ± 296	332 ± 61	Aerobic and anaerobic effluent (1:1)
1–3	27–32	Inhibition for AOB	2447 ± 422	510 ± 55	Aerobic and anaerobic effluent (1:2)
1–4	33–48	Activity recovery for AOB	767 ± 242	515 ± 189	Aerobic and anaerobic effluent (2:1)
2–1	49–52	Starting up for single–stage ANAMMOX	782 ± 162	143 ± 66	All aerobic
2–2	53–92	Increasing influent nitrogen load	522 ± 88	287 ± 146	4–6 dilution times of the anaerobic effluent
2–3	93–121	steady operation	844 ± 62	542 ± 106	2–3 dilution times of the anaerobic effluent

**Table 2:** Engineering application examples of single–stage ANAMMOX process.

Reference	Type of Influent	Influent COD (mg/L)	Influent NH_4_^+^–N (mg/L)	TN Removal Load (kg/m^3^/d)	TN Removal Rate (%)
[17]	Sludge digestion liquid	500	500	0.68	84%
[18]	Sludge digestion liquid	100	500	0.4	90%
[19]	Synthetic wastewater	−	300	0.73	73%
[20]	Reject water	1000	1000	0.4	90%
[21]	Synthetic wastewater	30–180	600	1.69	77%
This study	Pharmaceutical wastewater	400–1000	600	1.0	75.2%
